# Efficient quality control of platelet-rich plasma preparation using computer vision and deep learning

**DOI:** 10.1117/1.JBO.30.6.065003

**Published:** 2025-06-30

**Authors:** WangXiang Mai, WeiYi He, Rongchi Mo, GuoHao Liu, Jing Hong, WanYue Li, Li Luo, ZhuoMing Chen

**Affiliations:** a The First Affiliated Hospital of Jinan University, Department of Rehabilitation, Guangzhou, China; b The First Affiliated Hospital of Jinan University, Department of Pediatrics, Guangzhou, China; c Guangdong Zhaoqing Aviation Vocational College, Zhaoqing, China; d South China University of Technology, School of Mathematics, Guangzhou, China; e Guangdong University of Technology, School of Physics and Optoelectronic Engineering, Guangzhou, China

**Keywords:** platelet-rich plasma quality control, computer vision, deep learning

## Abstract

**Significance:**

Platelet-rich plasma (PRP) is a critical component in regenerative medicine, with applications in tissue repair and inflammation regulation. Consistent preparation quality is essential for therapeutic efficacy, but traditional quality control (QC) methods are labor-intensive, slow, and prone to variability.

**Aim:**

We introduce a computer vision–based automated PRP QC model using deep learning to improve the efficiency and accuracy of PRP preparation.

**Approach:**

Blood samples were collected and processed in the laboratory to prepare PRP. Images of the samples were manually captured. Medical-grade QC evaluations determined sample quality, which was labeled for model training. The image data were preprocessed and analyzed using a ResNet18 convolutional neural network combined with a binary classifier to develop a PRP QC model. Training and testing were conducted using data from patients, and the model’s accuracy was tested on the independent unavailable dataset.

**Results:**

The PRP QC model achieved an average classification accuracy of 82.5% on unavailable datasets (previously unseen test samples), significantly reducing the time required for QC to under 1 min.

**Conclusions:**

We demonstrate a nondestructive, real-time QC method for PRP preparation with computer vision and deep learning, offering a practical and scalable solution to improve clinical outcomes in regenerative medicine.

## 1 Introduction

Platelet-rich plasma (PRP) has emerged as a remarkable advancement within the realm of regenerative medicine.^1^^–^^5^ It is renowned for its capacity to drive tissue repair, modulate inflammation, and stimulate cellular proliferation. By extracting and concentrating platelets from autologous blood, PRP offers a natural and minimally invasive treatment modality across a diverse range of medical fields such as orthopedics,^6^^–^^11^ dermatology,^12^^,^^13^ and dentistry.^14^^–^^16^ Nevertheless, the clinical efficacy of PRP treatments is highly contingent upon the quality of the prepared PRP, which is influenced by factors, including platelet concentration, activation status, and the presence of bioactive growth factors. The variability in these factors, stemming from differences in preparation techniques, patient-specific characteristics, and procedural inconsistencies, has underscored the urgent need for reliable and standardized QC methods to guarantee consistent therapeutic outcomes.

Traditional QC approaches, exemplified by platelet counting and growth factor quantification,^17^^–^^19^ although effective, are beset with several limitations. These methods typically necessitate extensive laboratory infrastructure,^20^^–^^22^ are time-consuming (generally taking 20 to 60 min to complete),^23^^–^^25^ and rely heavily on specialized personnel.^20^^,^^21^ Consequently, they not only augment operational costs but also lead to inefficiencies. Moreover, they are often destructive in nature, as a portion of the PRP sample must be removed for testing, ^26^^,^^27^ thereby reducing the amount available for clinical application. This creates a conundrum where ensuring quality inadvertently diminishes the quantity of the usable product. With the escalating utilization of PRP in clinical practice, especially in the context of individualized treatment regimens, the constraints of conventional QC methods have spurred the pursuit of innovative alternatives.

The recent strides in deep learning^28^^–^^30^ and computer vision^31^^–^^34^ have proffered a promising substitute for traditional PRP QC methods. Computer vision empowers the automated analysis of visual features from high-resolution images, presenting a noninvasive and efficient means of evaluating biological samples. Deep learning models, such as convolutional neural networks (CNNs), including the residual network (ResNet), have demonstrated outstanding performance in image classification tasks.^35^^–^^40^ Their ability to learn hierarchical features via residual connections effectively mitigates the gradient vanishing problem in deep networks. These models have been successfully applied in a plethora of medical image analysis tasks.^41^^–^^45^ Integrating such models into the PRP QC process holds the potential to revolutionize the field by enabling rapid and reliable assessments of sample quality and reducing the reliance on traditional laboratory methods.

In this study, we have developed a novel computer vision–based system for the QC of PRP preparation. This system is predicated on the ResNet18 CNN in conjunction with a binary classification model. It analyzes the images captured during the preparation process to extract visual features such as color and transparency. After undergoing training and testing, it can efficiently classify PRP samples as either “qualified” or “unqualified”. The system is designed to provide a noninvasive and real-time QC solution that aligns with the demands of modern clinical practice. Our methodology combines a controlled imaging setup, advanced image preprocessing techniques, and state-of-the-art deep learning methods to comprehensively evaluate the system’s performance and ensure its robustness and accuracy.

This work has the potential to address several longstanding challenges in PRP preparation. First, it provides a standardized method for assessing PRP quality based on computer vision, reducing the variability induced by operator-dependent factors. Second, the nondestructive nature of the system preserves the integrity of PRP samples, ensuring that the entire volume is available for therapeutic application. Third, the rapid feedback provided by the system enables real-time adjustments to preparation parameters, guaranteeing optimal sample quality. Finally, by leveraging deep learning, the system is scalable and adaptable, with the potential to incorporate additional features and modalities in the future. This study contributes to the broader field of regenerative medicine and lays the foundation for further advancements in automated biomedical diagnostics. The results highlight the transformative potential of integrating artificial intelligence (AI) into clinical workflows, paving the way for more efficient and reliable healthcare solutions.

## 2 Research Methods

### 2.1 Sample Preparation

The research included a cohort of 55 patients, aged between 18 and 74 years, who received treatment at our medical facility from September 2022 to May 2023, primarily for rehabilitation of sports injuries and chronic musculoskeletal pain. The baseline characteristics of the participants are summarized in Table 1. Rigorous ethical protocols were strictly followed. Before their participation, all patients provided informed consent in writing. A comprehensive set of exclusion criteria was formulated to ensure the reliability and homogeneity of the samples. Patients with coagulopathy, active malignancies, anemia (hemoglobin levels below 120  g/L), or who had used antiplatelet or nonsteroidal anti-inflammatory drugs within the last three days were excluded; none of these patients were included in the final experiment. Moreover, female patients within three days of menstruation and pregnant individuals were ineligible. These exclusionary measures were essential for minimizing potential confounding factors that could affect the quality and characteristics of the blood samples used for PRP preparation.

**Table 1:** Baseline characteristics of participants (n=55).

Factor	Classification	Number (%)
**Sex**	Male	27 (49.1%)
	Female	28 (50.9%)
**Age**	18–29	8 (14.5%)
	30–44	10 (18.2%)
	45–59	16 (29.1%)
	60–74	21 (38.2%)
**BMI**	<18.5 (underweight)	5 (9.1%)
	18.5–23.9 (normal)	35 (63.6%)
	24–27.9 (overweight)	14 (25.5%)
	≥28 (obese)	1 (1.8%)

BMI = body mass index, calculated as weight in kilograms divided by height in meters squared ($\mathrm{kg}/m^{2}$).

**Fig. 1:**
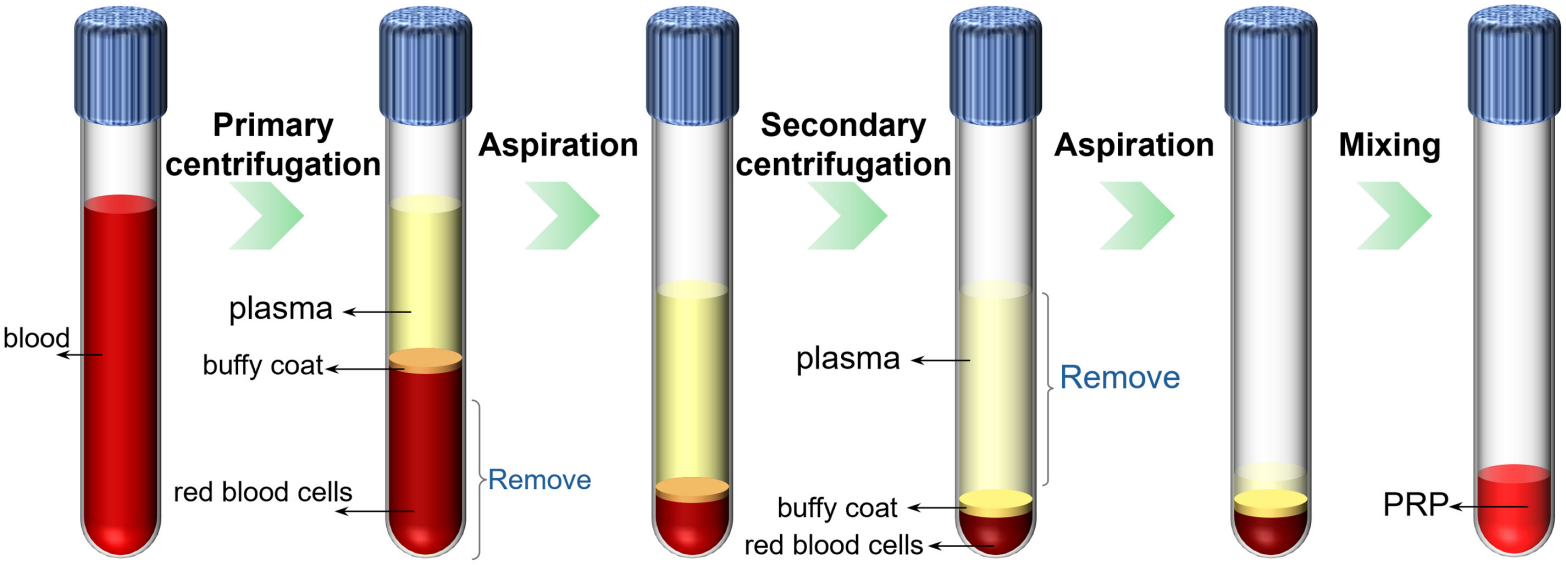
Schematic of the PRP preparation process, involving two-stage centrifugation, aspiration of specific layers, and final mixing to obtain PRP.

Under strict sterile conditions, 18 mL of venous blood was carefully drawn from each patient. To prevent coagulation, 2 mL of sodium citrate solution was added as an anticoagulant. The blood sample was then gently and thoroughly mixed to ensure uniform distribution of the anticoagulant. A standardized two-step centrifugation protocol was employed to prepare PRP, and the detailed preparation process is illustrated in Fig. 1. The actual preparation procedure is also described in the Supplementary Material. During the first centrifugation step, the blood sample was placed in a high-speed centrifuge (Model TD5A, rotor radius 11 cm) manufactured by Shanghai Luxiangyi Centrifuge Instrument Co., Ltd., Shanghai, China. The sample was centrifuged at 1500 rpm for 10 min. This initial centrifugation separated the blood into three distinct layers: the upper layer consisting of plasma, the middle layer (buffy coat), and the bottom layer containing red blood cells (RBCs). Approximately 3 mm of RBCs just below the buffy coat–RBC interface were retained, whereas the remaining excess RBCs were aspirated through the central channel of the centrifuge tube and discarded. The plasma layer, buffy coat, and part of the upper RBC layer were retained for further processing. Notably, retaining the uppermost portion of the RBC layer is essential as platelets, though primarily concentrated in the buffy coat, can partially migrate into the adjacent RBC layer due to gravitational settling. Over- or under-removal of the RBC layer may result in insufficient platelet recovery or suboptimal PRP concentration, ultimately leading to unqualified PRP. A detailed comparison between qualified and unqualified PRP samples is provided in Supplementary Material. In the second centrifugation step, the collected supernatant was centrifuged at a higher speed of 2000 rpm for 10 min. Following this, ∼85% of the upper plasma layer, which contains relatively fewer platelets, was carefully removed and discarded. This procedure resulted in ∼3  mL of concentrated PRP being prepared per patient.

Throughout the entire preparation process, utmost care was taken to maintain the sterility and integrity of the PRP samples. To validate the quality of the prepared PRP, standard laboratory techniques were utilized. Platelet counts were accurately determined using advanced hematology analyzers, and the activation status of platelets was evaluated through specialized assays. These laboratory results served as the gold-standard reference for the subsequent development and validation of the deep learning model.

### 2.2 Image Acquisition and Preprocessing

To capture high-quality and consistent images of blood samples, a meticulously designed imaging setup was established, ensuring precision and reproducibility throughout the process. The imaging system consisted of a calibrated illumination setup, a high-resolution digital camera, and a custom-engineered sample holder, all of which were optimized to minimize variability and maximize image clarity. The illumination system featured two parallel 15 W LED light panels with a color temperature of 5500 K. These panels were strategically mounted at the top of a closed imaging box to provide uniform and diffused lighting. This setup effectively minimized shadows on the sample tubes, although reflections on the curved surface of the tubes proved difficult to completely eliminate. To ensure stable brightness levels and consistent illumination, the lighting system was preheated for 10 min before each imaging session.

**Fig. 2:**
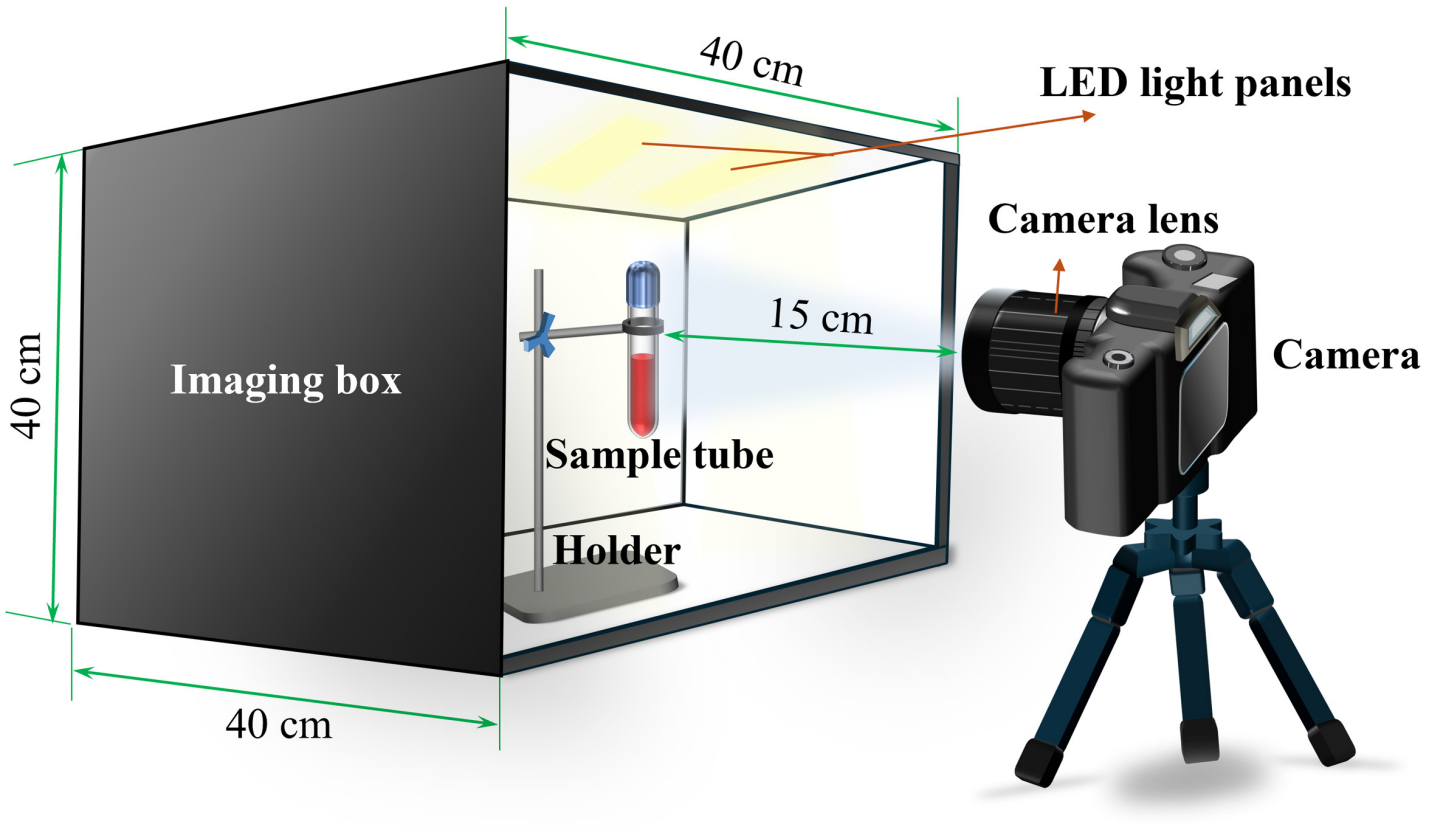
Schematic of the imaging system.

Images were captured using a Nikon D90 digital camera, selected for its stable and reliable imaging performance imaging performance. The D90 is equipped with an APS-C (DX-format) CMOS sensor measuring 23.6×15.8  mm, with a native resolution of 4288×2848  pixels (∼12.3  megapixels) and a pixel pitch of 5.5  μm. The camera was paired with a NIKKOR AF-S DX 18-105 mm f/3.5-5.6 G ED VR lens. As a mature and widely adopted imaging device, the D90 offers full manual (M mode) control over exposure parameters, ensuring image acquisition consistency. Although its performance in low-light conditions and fine-detailed reproduction may not match that of newer high-resolution models, its affordability, operational stability, and absence of in-camera optimization algorithms make it well-suited for standardized, static imaging required in this study. The camera was positioned at a fixed distance of 15 cm from the sample tubes to maintain consistent focal length and perspective. Through a series of preliminary tests, the camera settings were fine-tuned to achieve optimal image quality under the given conditions. The finalized parameters included a shutter speed of 1/400 s, an aperture of f/13, and an ISO sensitivity of 800. These settings provided a balanced exposure, adequate depth of field, and excellent clarity across all samples. To maintain consistent positioning during imaging, the sample tubes were placed upright in a custom-designed holder within the imaging box. This holder ensured that the tubes were aligned at a uniform height relative to the camera lens, minimizing positional variability among samples. Figure 2 provides a detailed schematic of the imaging system employed in this study, with the major commercially available components summarized in Table 2. Images were acquired at two critical stages of the PRP preparation process. First, baseline images of whole blood were taken before centrifugation to establish a reference for comparison. Second, after the second-stage centrifugation and mixing processes were completed, additional images were captured to document the prepared PRP. This systematic imaging approach ensured the acquisition of high-resolution, reliable visual data critical for the accurate evaluation of blood sample properties.

**Table 2:** Key components of the imaging system and their commercial specifications.

No.	Component	Model/specification	Manufacturer (company, city, and country)
**1**	Camera	Nikon D90	Nikon Optical Instruments (China) Co., Ltd., Wuxi, Jiangsu, China
**2**	Camera Lens	NIKKOR AF-S DX 18–105 mm f/3.5–5.6G ED VR	Nikon Optical Instruments (China) Co., Ltd., Wuxi, Jiangsu, China
**3**	Imaging Box	40 × 40 × 40 cm	Shanghai Meinuo Photography Equipment Co., Ltd., Shanghai, China
**4**	LED light panels	2 × 15 W; Color temperature: 5500 K; Illuminance: 1200 lux ±5%; uniformity >90%	Shanghai Meinuo Photography Equipment Co., Ltd., Shanghai, China
**5**	Holder	Base: 16 × 9.3 cm, Tube rack height: 30 cm	Jiangsu Wangsen Laboratory Instruments Co., Ltd., Taizhou, Jiangsu, China

The raw images obtained from the camera underwent a series of pre-processing steps to optimize their quality for deep learning–based analysis. The first step in the process involved precise cropping to isolate the blood sample regions, removing extraneous background elements. This not only minimized computational noise but also focused the model’s attention on the relevant sample area, thereby enhancing the efficiency of the analysis. To mitigate the challenge of a limited dataset and improve model robustness, a diverse set of data augmentation techniques was applied. These included random rotations of up to ±15  deg, simulating possible variations in tube orientation during the imaging process; slight horizontal and vertical translations to account for any potential misalignments; and horizontal flips to ensure that the model was invariant to the sample’s orientation. By applying these transforms, 100 subtly varied images were generated from each original blood sample image, resulting in a total dataset of 5500 images. These augmented images introduced fine-grained differences while maintaining the essential characteristics of the originals, thereby improving the model’s generalization to real-world variations in sample positioning and enhancing training performance without compromising image quality or clinical relevance.

**Fig. 3:**
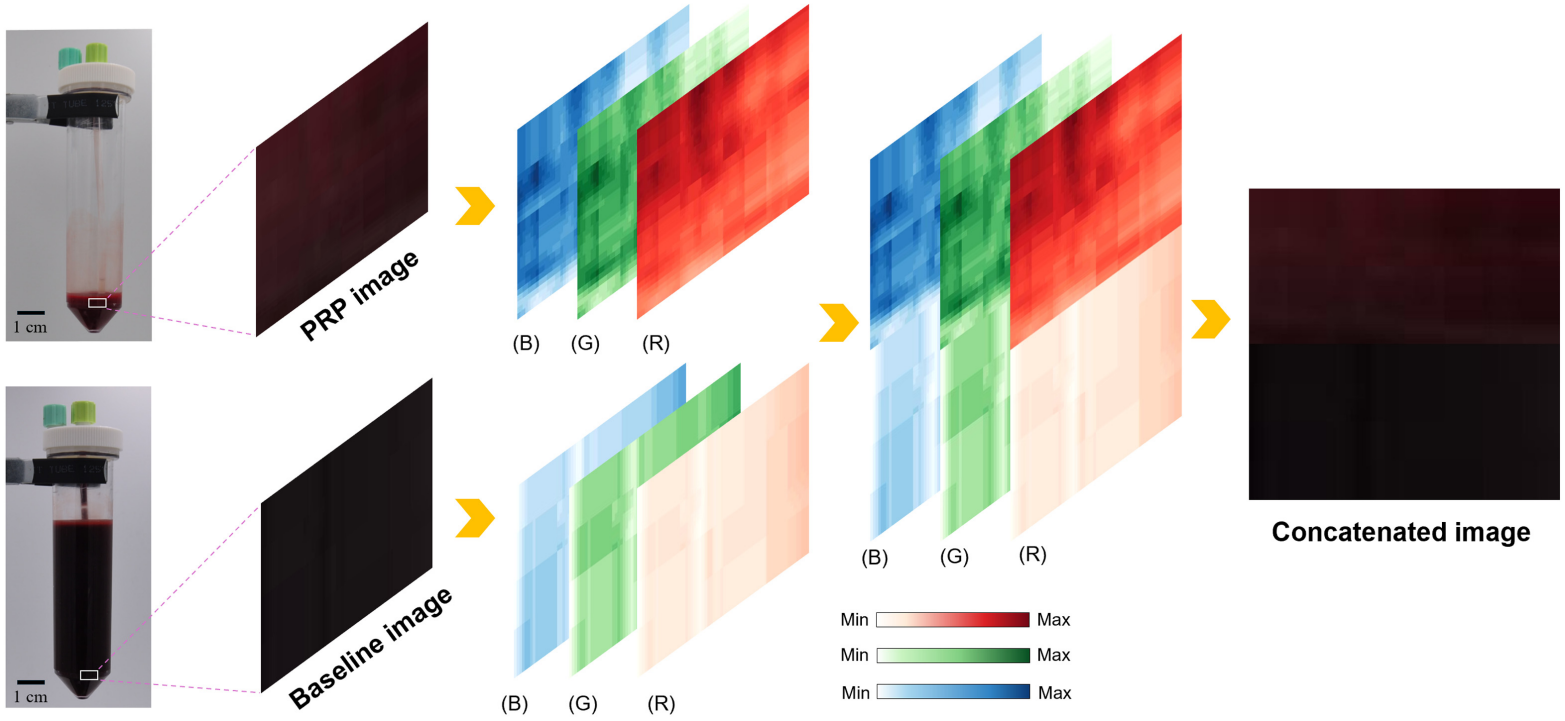
Sample image processing for the PRP QC model. Baseline and PRP images were obtained from whole blood (before centrifugation) and the prepared PRP, respectively. They were concatenated into 64×64  pixel input image. Panels (R), (G), and (B) represent the red, green, and blue channel values at each pixel position, with color intensity directly proportional to the pixel values.

To ensure compatibility with the ResNet18 CNN model and preserve key visual features, both the baseline images (pre-centrifugation) and PRP images were resized to 32×64  pixels. These images, captured from approximately the same region of the tube as shown in Fig. 3, were then concatenated into 64×64  pixel input samples, preserving the temporal progression from whole blood to PRP while minimizing individual variations, as illustrated in the image concatenation process shown in Fig. 3. As the blood samples were collected from venous blood, which is characteristically dark red due to its high deoxygenated hemoglobin content, both the baseline and PRP images naturally appeared relatively dark. To better detect and compare the subtle color variations during the PRP preparation process, we analyzed the RGB channels of the images, which provided a more sensitive and quantitative representation of the visual changes.

Compared with the baseline image, the PRP image exhibited a significant increase in R channel intensity, along with moderate rises in the G and B channels. This shift is primarily attributed to the removal of RBCs during PRP preparation. RBCs contain hemoglobin, which strongly absorbs light, particularly in the red spectrum. As the RBC concentration decreases, less red light is absorbed and more is reflected, resulting in higher R channel values and a visibly redder image. A similar trend is observed in the G and B channels: the reduction in RBCs leads to decreased absorption and increased reflection of green and blue light, producing a moderate increase in their respective intensities. Although platelet concentration increases in PRP, its influence on the overall spectral profile is relatively minor compared with the effect of RBCs. Overall, the decrease in RBC concentration is the dominant factor driving the changes in RGB composition, shifting image tones from deep dark red to a marginally brighter dark red. These subtle yet consistent changes are clearly illustrated in the concatenated image shown in Fig. 3, which capture the color transformation resulting from PRP processing.

### 2.3 Model Development

The model development and training were performed using Python programming language. To improve the training efficiency of the model and accelerate convergence, all image pixel values were normalized to the range of 0 to 1 through division by 255. In addition, each concatenated image was assigned a label based on laboratory-determined QC results. Specifically, samples meeting predefined criteria for platelet concentration and activation, established through rigorous medical-grade assays, were classified as “qualified” (class 1). Samples failing to meet these criteria were labeled as “unqualified” (class 0). Based on patient-level QC assessments of PRP samples from 55 patients, 32 were classified as qualified and 23 as unqualified. The final dataset included 5500 sample images, with 3200 images (∼60%) labeled as class 1 and 2300 images (∼40%) as class 0. These labels provided the ground truth for both training and validating the deep learning model, ensuring that the final model could accurately classify blood samples based on quality.

**Fig. 4:**
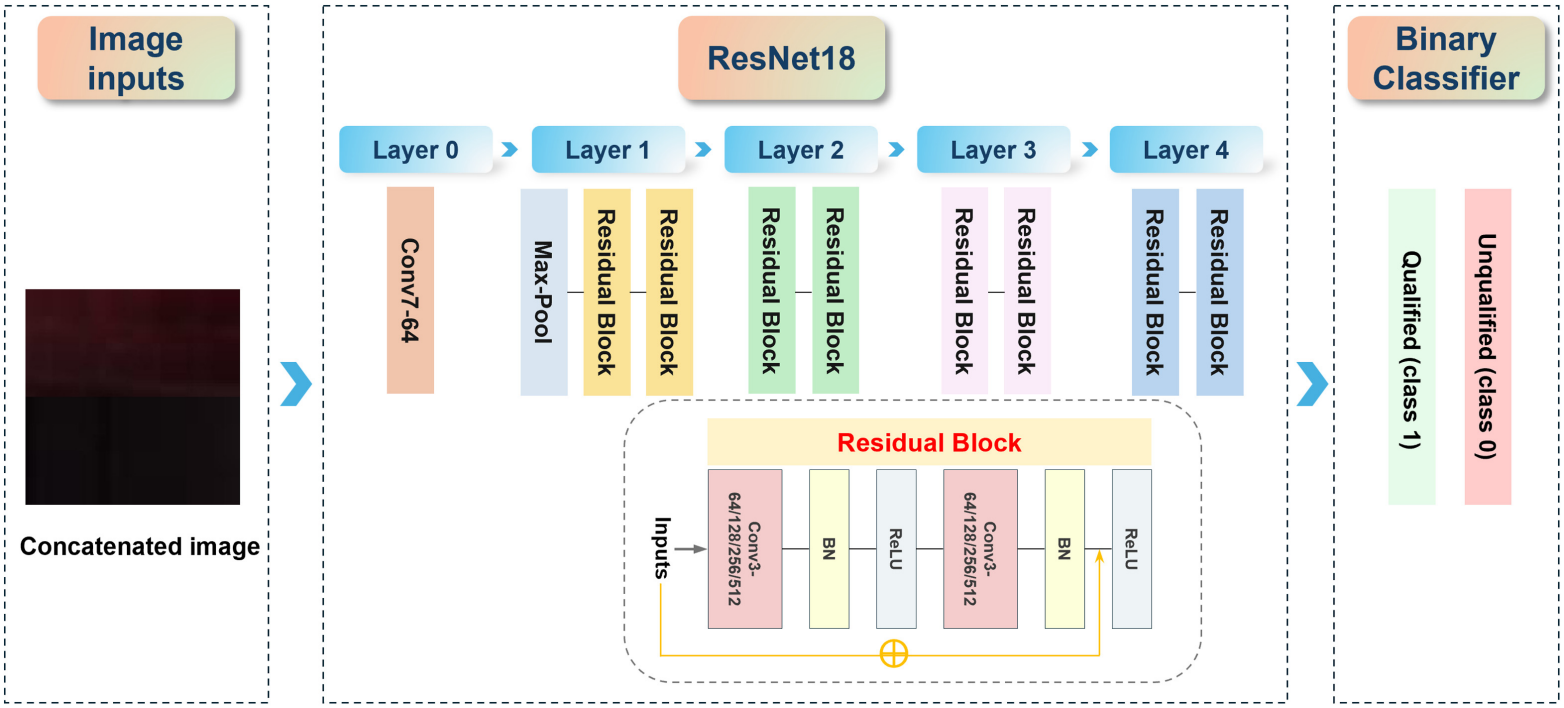
PRP QC model framework, based on ResNet18 and a binary classification structure.

The PRP QC model used in this study is based on ResNet18 and consists of a binary classification framework, as shown in Fig. 4. ResNet18 is a highly efficient CNN known for its strong performance in image classification tasks. It incorporates residual connections, which address the vanishing gradient problem, allowing for stable training of deeper networks and the extraction of complex features from images.

The ResNet18 begins with an initial convolutional layer that applies a 7×7 convolutional kernel to extract fundamental features from the input images. This is followed by a max-pool layer, which reduces the spatial dimensions of the feature maps, thereby decreasing computational complexity while retaining critical information. This preprocessing step prepares the data for subsequent processing. The network’s core consists of eight residual blocks, each containing two convolutional layers. These layers are equipped with batch normalization (BN) and rectified linear unit (ReLU) activation functions. BN normalizes the input to each layer, improving training stability and accelerating convergence, whereas the ReLU activation introduces nonlinearity, allowing the model to learn complex patterns within the data. These layers work together to progressively extract more abstract and hierarchical features, thereby building a comprehensive representation of the input images. At the final stage, the network utilizes a global average pooling layer, which aggregates the feature maps into a single representative vector. This vector is then passed through a fully connected layer to produce the output of the binary classifier (BC). Binary cross-entropy loss was selected as the objective function as it effectively quantifies the divergence between predicted probabilities and actual labels, making it particularly suitable for binary classification tasks.

### 2.4 Dataset-Splitting Strategy

To facilitate model optimization and evaluation, the complete dataset of 5500 concatenated images, each derived from 55 patients with 100 images per patient, was split into training and testing subsets at an approximate 80:20 ratio. Two distinct data-splitting strategies were designed based on whether potential correlations exist between the training and testing sets at the patient level: (1) available dataset (random sample split) and (2) unavailable datasets (patient-independent split).

In the available dataset strategy, all 5500 images were randomly partitioned at the sample level, resulting in 3200 training images and 2300 testing images. As each patient contributed 100 images from different cropped regions, images from the same patient could appear in both subsets. Although individual images differ, this overlap introduces patient-level correlations between the training and testing sets. Consequently, the testing set may contain patient-specific features already encountered during training, making it a nearly available dataset.

By contrast, the unavailable dataset strategy involved splitting the data at the patient level to ensure independence between training and testing sets. Specifically, samples from 45 patients (4500 images) were used for training, whereas samples from the remaining 10 patients (1000 images) were reserved for testing. To maintain a consistent class distribution (∼60:40 ratio of qualified to unqualified), the training set comprised 26 qualified and 19 unqualified patients (2600 and 1900 images, respectively), whereas the testing set included 6 qualified and 4 unqualified patients (600 and 400 images, respectively). This strategy ensures that the model evaluates generalization performance on previously unseen patients.

Model optimization and feasibility evaluation were primarily performed using the available dataset. The model was fine-tuned on this dataset to improve its practical applicability. Key hyperparameters were carefully optimized, including the use of the Adam optimizer with a learning rate of 0.001 and weight decay of 0.0001 for preventing overfitting and making regularization.

During training, a batch size of 32 was used to balance computational efficiency and sensitivity to data variation. The model’s weights were updated iteratively to minimize the loss. To prevent overfitting and enhance generalization, early stopping was employed, terminating training if no improvement in validation loss was observed over 30 consecutive epochs. In the testing phase, a smaller batch size of 16 was used to improve computational efficiency. To evaluate model robustness under varying data splits, multiple rounds of training and testing were conducted. Performance metrics were averaged across runs to obtain reliable assessments of model accuracy, stability, and generalization capability.

## 3 Results and Discussion

### 3.1 Model Performance

#### 3.1.1 PRP QC capabilities on the available dataset

**Fig. 5:**
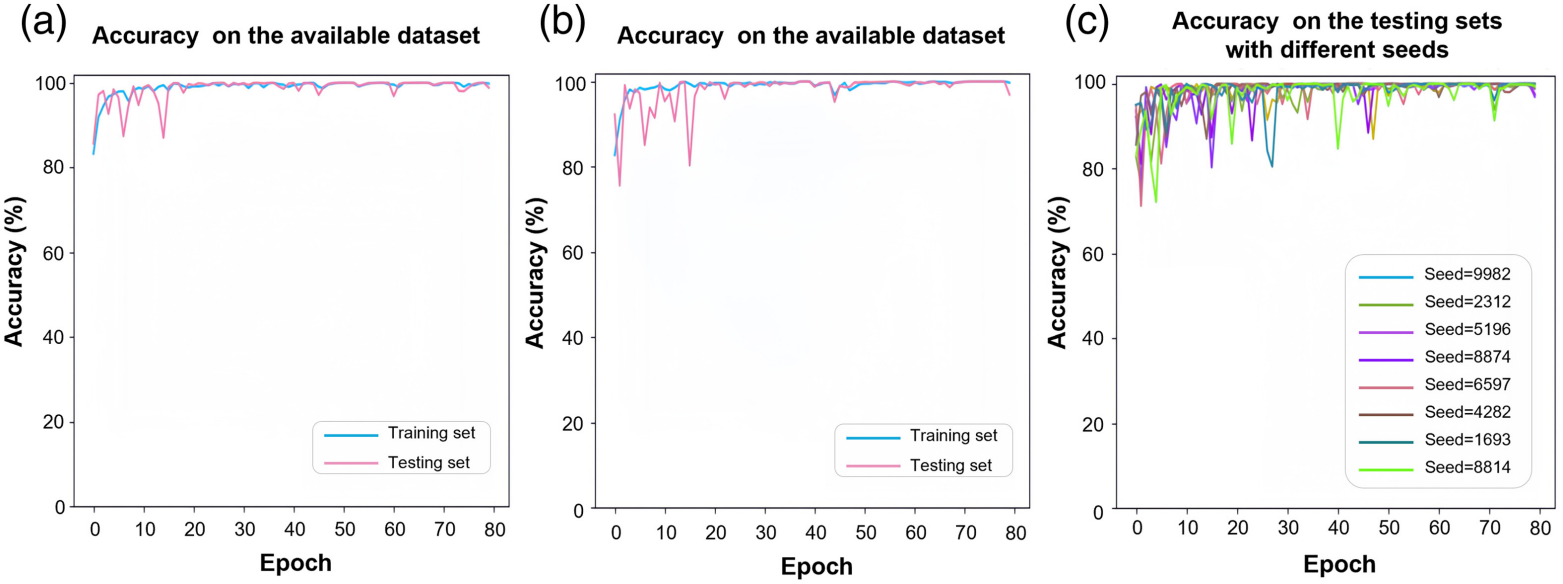
Performance evaluation of the PRP QC model on the available dataset under various data-splitting schemes. Panels (a) and (b) show the model’s accuracy on the training and testing sets under two distinct data-splitting schemes. Panel (c) presents the model’s performance across eight different data-splitting schemes on the available dataset.

The feasibility and performance of the PRP QC model were rigorously evaluated using the available dataset-splitting strategy. By applying two different random seeds, the dataset was split at the sample level to generate two distinct sets of training and testing data. Under both seed-based data splitting schemes, we first trained the model using the training sets. After each epoch, both the training and testing sets were fed into the model for classification to evaluate accuracy, as indicated by the blue and red curves in Figs. 5(a) and 5(b), respectively. Accuracy reflects the overall correctness of the model’s classifications and is defined as

$$\text{Accuracy}=\frac{\mathrm{TP}+\mathrm{FN}}{\mathrm{TP}+\mathrm{TN}+\mathrm{FP}+\mathrm{FN}}\times 100\%,$$ (1)

where TP, TN, FP, and FN denote the numbers of true positives, true negatives, false positives, and false negatives, respectively.

During the first 20 epochs, the PRP QC accuracy on the training set steadily improved. However, during the early epochs, the accuracy of the testing set exhibited instability, fluctuating among (90±10)% until stabilizing at 100% around the 30th epoch. This trend underscores the model’s capacity to effectively learn PRP-specific features and accurately distinguish between qualified and unqualified samples. The high separability observed in the feature space suggests that each category possesses consistent intra-class features and distinct inter-class characteristics. Minor fluctuations in later-stage accuracy may be attributed to overfitting caused by the dataset’s limited size or model complexity. Nonetheless, the selected model configuration, achieving consistent 100% accuracy near the 30th epoch, demonstrates strong robustness and optimal performance.

To minimize the potential influence of incidental bias arising from individual data-splitting schemes, additional variability was introduced into the evaluation process. Specifically, eight different random seeds were selected, corresponding to eight distinct data-splitting schemes on the available dataset, to further observe the model’s performance on the testing sets. The results of these analyses are shown in Fig. 5(c). The model consistently reached ∼100% testing accuracy around the 30th epoch across all data-splitting schemes. The high accuracy derives from its effective use of convolutional and residual layers based on ResNet18 to extract key visual features essential for PRP quality classification. The outstanding performance indicates that localized sample images can reliably reflect the overall quality of prepared PRP, highlighting the model’s robustness, reliability, and practical applicability in real-world PRP quality control.

#### 3.1.2 Generalization ability of the model

To evaluate the model’s generalization capability, the dataset was split at the patient level (i.e., using the unavailable dataset-splitting strategy). This partition ensured that, prior to testing, the model had not been exposed to any similar images from the same patients, thereby enabling a robust and unbiased evaluation of its generalization performance on truly unseen data.

**Fig. 6:**
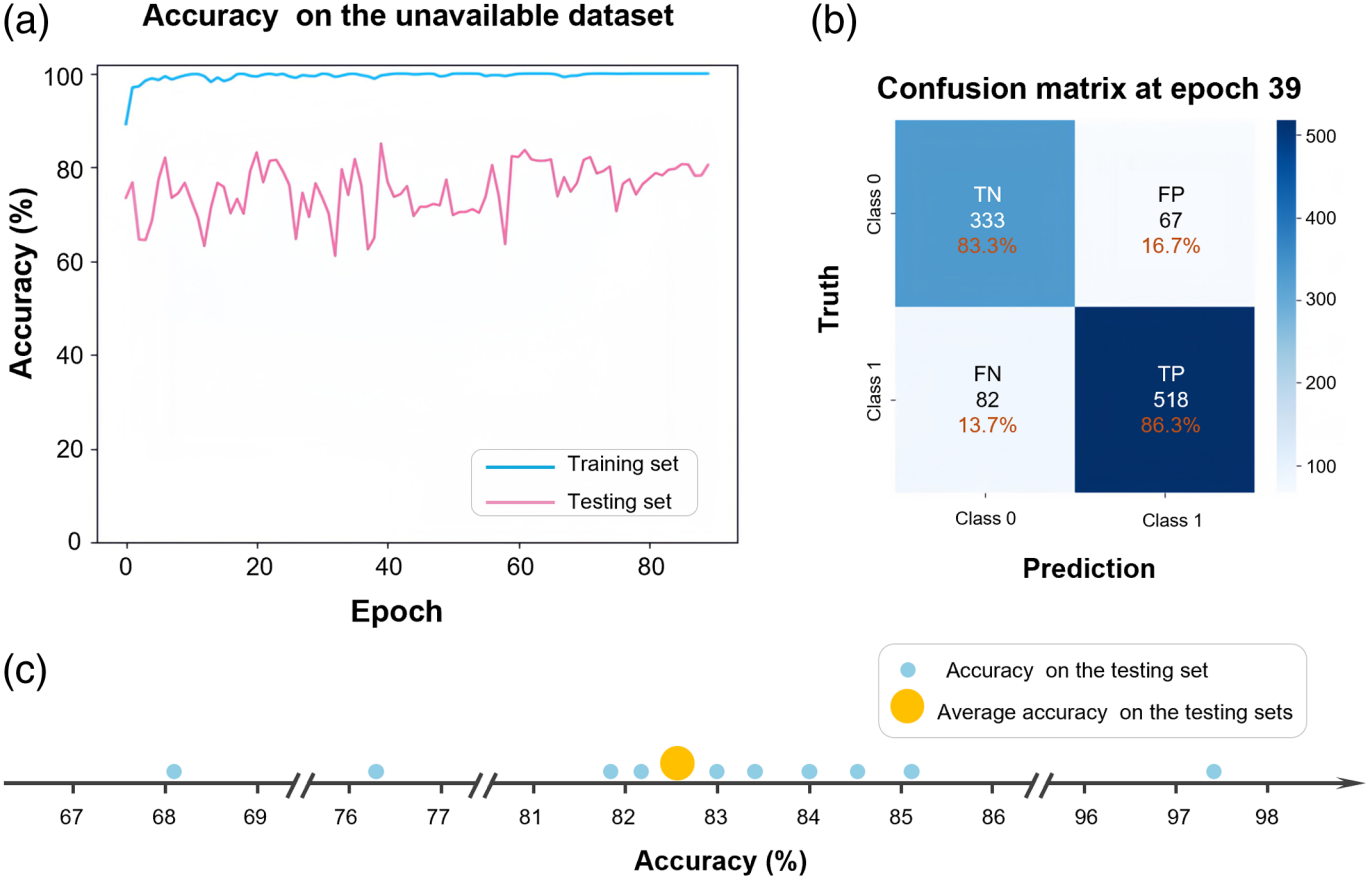
Performance evaluation of the PRP QC model on the unavailable dataset. (a) Accuracy trends for the training and testing sets under a representative data split on the unavailable dataset. (b) Confusion matrix of testing set in panel (a) at epoch 39. (c) Quantitative analysis of the model’s performance across 10 independent tests on the unavailable dataset, each using a different seed to realize dataset partition. The accuracy of each independent testing is represented by blue dots, whereas the average testing accuracy is represented by yellow dot.

Based on a fixed feature extractor, BC, and parameter configuration, we evaluated the model’s accuracy on the unavailable dataset. Figure 6(a) illustrates the accuracy trends in the training and testing sets under a representative seed-based data splitting scheme. As expected, the model quickly converged to 100% accuracy on the training set, further validating our earlier findings that the model effectively extracts features from localized image samples and reliably distinguishes between qualified and unqualified PRP samples. In the testing set, the model’s accuracy initially improved with training but then entered a state of fluctuation. These fluctuations may be influenced by factors such as the quality and quantity of the sample data, model architecture choices, and parameter settings during training. Despite these variations, the model achieved a peak accuracy of 85.1% at epoch 39 under this specific data split. As shown in Fig. 6(b), the confusion matrix provides a detailed view of the model’s classification results. The model correctly identified 518 qualified samples (true positives, TP) and 333 unqualified samples (true negatives, TN). Meanwhile, it misclassified 67 unqualified samples as qualified (false positives, FP) and 82 qualified samples as unqualified (false negatives, FN). The true positive rate (TPR), true negative rate (TNR), false negative rate (FNR), and false positive rate (FPR) are defined as

$$\mathrm{TPR}=\frac{\mathrm{TP}}{\mathrm{TP}+\mathrm{FN}}\times 100\%,$$ (2)

$$\mathrm{TNR}=\frac{\mathrm{TN}}{\mathrm{TN}+\mathrm{FP}}\times 100\%,$$ (3)

$$\mathrm{FNR}=\frac{\mathrm{FN}}{\mathrm{TP}+\mathrm{FN}}\times 100\%,$$ (4)

$$\mathrm{FPR}=\frac{\mathrm{FP}}{\mathrm{TN}+\mathrm{FP}}\times 100\%.$$ (5)

Based on the above, the TPR, TNR, FNR, and FPR are 86.3%, 83.3%, 13.7%, and 16.7%, respectively. These results indicate that the model maintains balanced performance across both classes, with a slightly stronger capability in recognizing qualified samples. The relatively low FPR and FNR further demonstrate its robustness and potential for practical application in PRP quality control.

To quantitatively assess the model’s accuracy under the unavailable dataset, we conducted several tests with different seed-based data splitting schemes, as shown in Fig. 6(c). Specifically, we performed 10 independent tests, each with a different dataset partition, and additional details are provided in the Supplementary Material. Across these experiments, the model achieved an average testing accuracy of ∼82.5%, with individual results as follows: 85.1%, 81.8%, 76.2%, 84.5%, 83.0%, 83.3%, 84.0%, 97.3%, 68.1%, and 82.2%. This consistent performance further underscores the model’s ability to generalize to new data, despite some variability influenced by data partitioning and sample characteristics.

### 3.2 Advantages and Limitations

The PRP QC model provides substantial advantages for PRP QC compared with traditional methods, with one of its most notable strengths being its efficiency. Traditional techniques, such as platelet counting and growth factor detection, are generally time-consuming. These methods involve manual operation of equipment, platelet counting, and the use of specific reagents and instruments for growth factor analysis, all of which are time-consuming. By contrast, the ResNet18-based model completes the entire QC process in less than 1 min, significantly improving clinical workflow efficiency. This rapid turnaround allows healthcare professionals to swiftly assess PRP sample quality, enabling timely clinical decisions. Consequently, the risk of sample degradation due to prolonged waiting times is reduced, and PRP samples can be preserved and applied more promptly, optimizing the overall use of medical resources.

The model also offers significant improvements in accuracy, consistency, and nondestructiveness. Traditional methods heavily rely on human intervention, which introduces the potential for operator variability, leading to inconsistent results. For instance, differences in counting techniques or subjective interpretation of microscope images can result in discrepancies in platelet counts, whereas variations in reagent volumes or reaction times can affect growth factor testing outcomes. Furthermore, these methods often consume part of the sample and require reagent-based processing, which alters or even depletes the sample being tested. By contrast, the ResNet18-based model operates in a nondestructive manner by directly analyzing captured images of the PRP samples without altering them. This allows the entire sample to be preserved for therapeutic use. In addition, by automating the image analysis process and leveraging CNNs for feature extraction, the model ensures a high degree of consistency and reproducibility in QC tasks while significantly reducing subjective bias. For example, the model’s classification results for the same PRP sample are highly consistent across multiple tests, whereas human evaluations may vary. This consistency ensures that the QC process remains both accurate and dependable.

Moreover, the model provides real-time feedback during the PRP preparation process, allowing operators to immediately adjust the process based on the model’s output. For example, if platelet aggregation is detected as abnormal, the model can prompt the operator to adjust the centrifuge speed or time to optimize platelet separation. Similarly, if excessive impurities are identified in the sample, the operator can modify the sample collection tools or adjust pre-processing steps to improve the PRP sample quality. This real-time feedback loop ensures the continuous optimization of the PRP preparation process, leading to higher sample quality and more effective clinical outcomes.

However, despite these advantages, the model does have some limitations that need to be addressed. One significant limitation is its dependence on the dataset. Although data augmentation techniques help alleviate the issue of a small dataset, the model’s performance remains constrained by the quality and diversity of the training data. The performance of the PRP QC model is also highly sensitive to the quality of the input images. Factors such as uneven lighting during image acquisition can lead to overly bright or dark areas, making it difficult for the model to extract accurate features from those regions. In addition, image blurring can obstruct the model’s ability to identify fine details, which negatively impacts classification accuracy. Furthermore, the model is optimized for images captured with specific devices, featuring particular resolutions and color modes. When applied to images obtained from other devices with different characteristics, such as lower resolution, the model may struggle to detect subtle features and may require further adjustments or fine-tuning to maintain its performance.

## 4 Conclusion

In this study, we developed a model for the QC of PRP preparation. The model achieved an average accuracy of 82.5% on the unavailable dataset, significantly outperforming traditional methods in terms of efficiency and reducing the QC time to under 1 min. It provides a nondestructive, automated solution, ensuring consistency while minimizing operator-related variability. However, the system has some limitations. The model’s performance is dependent on image quality, and the relatively small dataset may affect its generalizability. In addition, the specialized imaging setup limits its accessibility. Despite these limitations, our work lays the foundation for future advancements in PRP QC. Future research will focus on enhancing the model’s robustness, expanding the dataset, and developing more accessible setups. With further improvements, this technology has the potential to be widely adopted in regenerative medicine, standardizing PRP preparation and ultimately improving clinical outcomes for patients.

## Supplementary Material

10.1117/1.JBO.30.6.065003.s01

## Data Availability

Data underlying the results presented in this paper are not publicly available at this time but may be obtained from the authors upon reasonable request.
