# Supplementary material for: Efficient quality control of platelet-rich plasma preparation using computer vision and deep learning
Source: J Biomed Opt. 2025 Jun 30;30(6):065003. doi: 10.1117/1.JBO.30.6.065003 (PMC12322746; doi:10.1117/1.JBO.30.6.065003)
Supplement: Supplementary file 1 [file JBO_030_065003_SD001.docx]

**Supplementary Materials for**

**“Efficient Quality Control of Platelet-Rich Plasma Preparation Using Computer Vision and Deep Learning”**

**WangXiang Mai,^a, †^ WeiYi, He^b, †^ Rongchi Mo,^c, †^ GuoHao Liu,^d^ Jing Hong,^a^ WanYue Li,^a^**

**Li Luo,^*, e^ ZhuoMing Chen^*, a^**

^a^Department of Rehabilitation, The First Affiliated Hospital of Jinan University, Guangzhou, Guangdong, China

^b^Department of Pediatrics, The First Affiliated Hospital of Jinan University, Guangzhou, Guangdong, China

^c^ Guangdong Zhaoqing Aviation Vocational College, Zhaoqing, China

^d^School of Mathematics, South China University of Technology, Guangzhou, Guangdong, China

^e^School of Physics and Optoelectronic Engineering, Guangdong University of Technology, Guangzhou, China

^* Address all correspondence to Li Luo, luoliphys@gdut.edu.cn; ZhuoMing Chen, zm120tchzm@qq.com^

^† Authors made equal contributions to this paper^

**S1. Actual Procedure for PRP Preparation**

**S2. Comparison Between Qualified and Unqualified PRP Samples**

**S3. Confusion Matrices Under Different Seeds in the Unavailable Dataset**

**S4. Performance Comparison Across Different Classifiers**

**S5. Performance Comparison of Feature Extraction Using ResNet18 and PCA**

**S1. Actual Procedure for PRP Preparation**

To complement the schematic illustration provided in the main text, the actual platelet-rich plasma (PRP) preparation process is presented here through real photographs, following the standardized two-step centrifugation protocol. Each step is labeled in correspondence with Fig. S1:

(a) Venous blood collection from the cubital vein under strict sterile conditions using a syringe.

(b) Transfer of the collected blood into a centrifuge tube preloaded with sodium citrate anticoagulant (2 mL for every 18 mL of blood).

(c) Blood sample after transfer, showing complete mixing with the anticoagulant.

(d) First centrifugation at 1500 rpm for 10 minutes in a high-speed centrifuge (Model TD5A, rotor radius 11 cm).

(e) Blood sample after the first centrifugation, showing three distinct layers: plasma, buffy coat, and red blood cells (RBCs).

(f) Retention of approximately 3 mm of red blood cells located just below the buffy coat-RBC interface, while excess RBCs were aspirated through the central channel of the centrifuge tube for removal.

(g) Blood sample after most of the RBCs have been removed, retaining the plasma, buffy coat, and upper RBC portion for platelet enrichment.

(h) Second centrifugation of the retained supernatant at 2000 rpm for 10 minutes to concentrate platelets.

(i) Blood sample after the second centrifugation, showing a more densely packed RBC sediment and a clearer plasma layer.

(j) Aspiration of the upper ~85% plasma layer, which contains fewer platelets.

(k) Blood sample after plasma removal, leaving the lower plasma fraction rich in platelets.

(l) Gentle mixing of the remaining content to resuspend the platelets uniformly, yielding the final PRP.


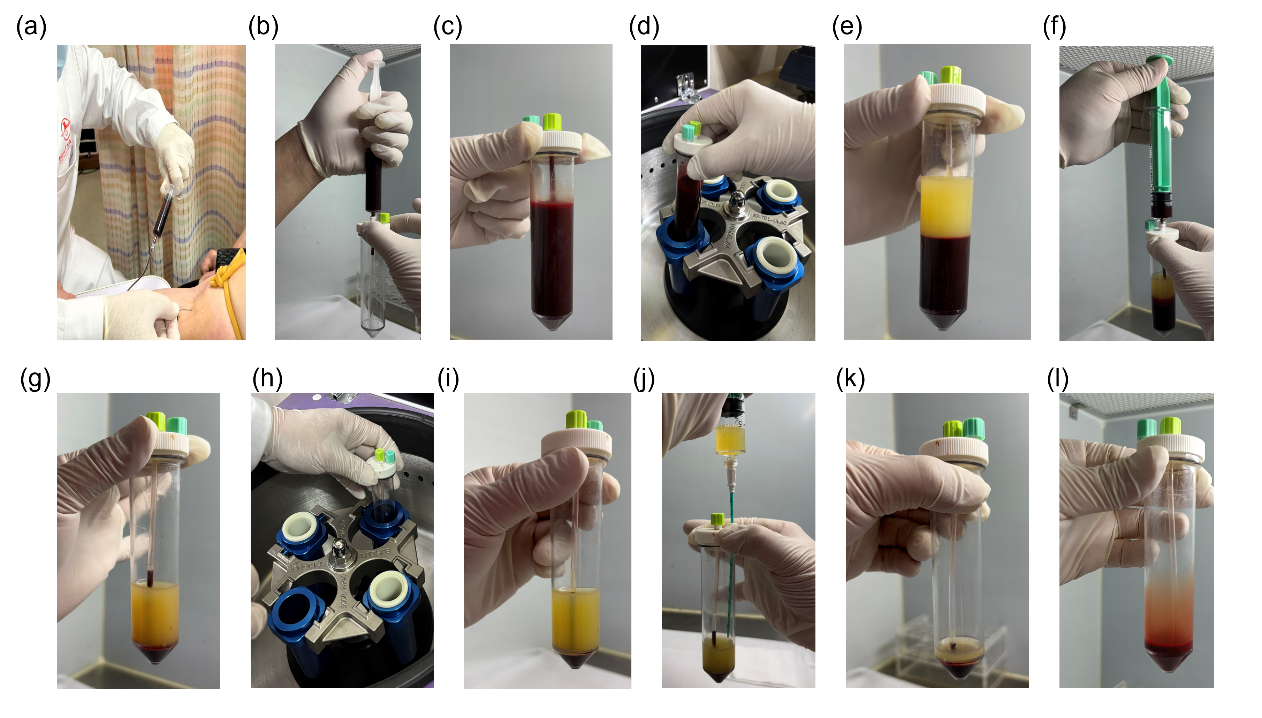


**Fig. S1** Photographic Representation of the PRP Preparation Process. (a) Venous blood collection from the cubital vein. (b) Transfer of whole blood into a centrifuge tube. (c) Blood sample after transfer. (d) First centrifugation step. (e) Blood sample after the first centrifugation. (f) Aspiration of the bottom portion of the RBC layer. (g) Blood sample after most of the RBCs have been removed. (h) Second centrifugation step. (i) Blood sample after the second centrifugation. (j) Aspiration of the upper plasma layer. (k) Blood sample after plasma removal. (l) Gentle mixing of the remaining content to obtain the final PRP.

**S2. Comparison Between Qualified and Unqualified PRP Samples**

Fig. S2 provides a visual comparison between qualified and unqualified PRP samples before and after preparation. In panel (a), the left image shows the whole blood sample prior to preparing a qualified PRP, and the right image presents the resulting PRP, which appears deep red in color. In panel (b), the left image displays the whole blood sample used to prepare an PRP which is unqualified, while the right image shows the final PRP. Compared to the qualified sample, the unqualified PRP appears brighter red. This brighter appearance is primarily due to excessive aspiration of the lower RBC layer after the first centrifugation step. Removing too many RBCs reduces light absorption and increases light reflection, making the sample visually lighter. However, because some platelets, despite being mainly concentrated in the buffy coat, can migrate into the upper RBC layer due to gravity, retaining this portion is essential. Over- or under-removal of RBCs may lead to insufficient platelet recovery or suboptimal PRP concentration, ultimately resulting in unqualified PRP. It is important to note that visual appearance alone is not sufficient to determine whether a PRP sample is qualified; objective analysis is required for accurate assessment.


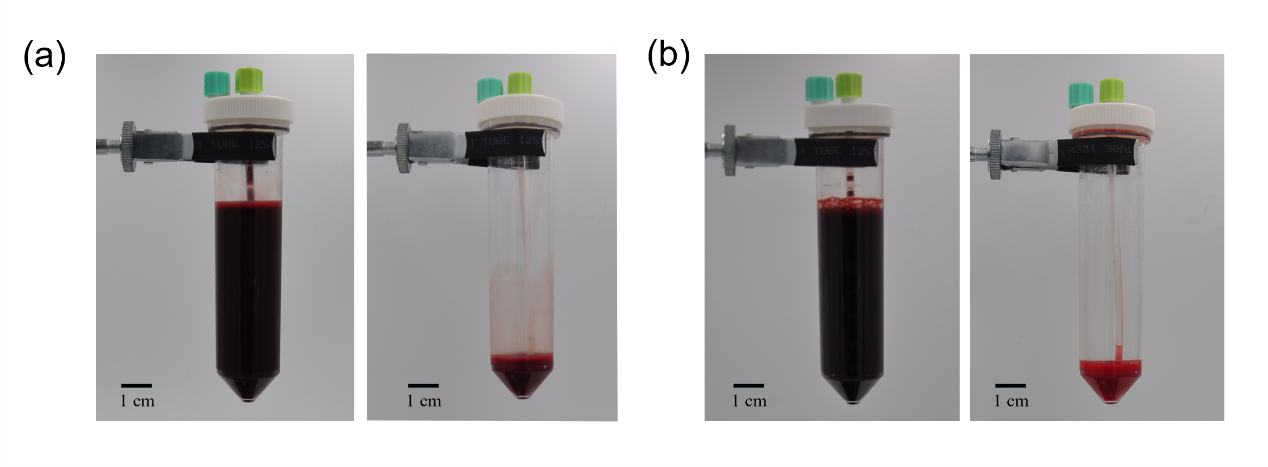


**Fig. S2** Comparison Between Qualified and Unqualified PRP Samples. (a) Left: Whole blood sample prior to preparation of the qualified PRP. Right: Image of qualified PRP.

(b) Left: Whole blood sample prior to preparation of the unqualified PRP. Right: Image of unqualified PRP.

**S3. Confusion Matrices Under Different Seeds in the Unavailable Dataset**

To quantitatively assess the model’s accuracy on the unavailable dataset, we conducted multiple tests using different seed-based data splitting schemes, as described in the main text. Specifically, we performed ten independent tests, each corresponding to a different dataset partition. One representative test with accuracy 85.1% is presented in detail in Fig. 6(a) and 6(b) of the main text. The remaining tests, using random seeds 2339, 3186, 4149, 5977, 5981, 7062, 6498, 7230, and 9242, whose accuracy are 81.8%, 76.2%, 84.5%, 83.0%, 83.3%, 84.0%, 97.3%, 68.1%, and 82.2%, and their confusion matrices are summarized in Fig. S3. These confusion matrices illustrate the model’s classification performance on the testing set under various initialization conditions.

Across all seeds, the model consistently achieves high true positive rates (TPR), particularly in seeds such as 6498, 9242, and 5977, where TPR exceeds 94%. This indicates the model’s strong ability to correctly identify qualified samples (Class 1). Meanwhile, the true negative rates (TNR) also remain reasonably high, especially under seed 6498 (96.5%), demonstrating the model’s capacity to correctly identify unqualified samples (Class 0).

Notably, considerable variation is observed across different seed. For instance, under seed 7230, the model's performance deteriorates significantly, with a true positive rate (TPR) of 71.5% and a true negative rate (TNR) of 63.0%. In contrast, under seed 6498, the model achieves an accuracy of 97.3%, with a TPR of 98.0% and a TNR of 96.3%. These results highlight the critical importance of feature representativeness in the training set under certain data partitioning schemes, as it can substantially impact classification performance during testing.

Despite these fluctuations, the false positive rate (FPR) and false negative rate (FNR) generally remain low across most seeds, underscoring the overall robustness and generalizability of the model in classifying PRP quality. These results further support the feasibility of employing the proposed model in real-world quality control applications where data availability is limited.


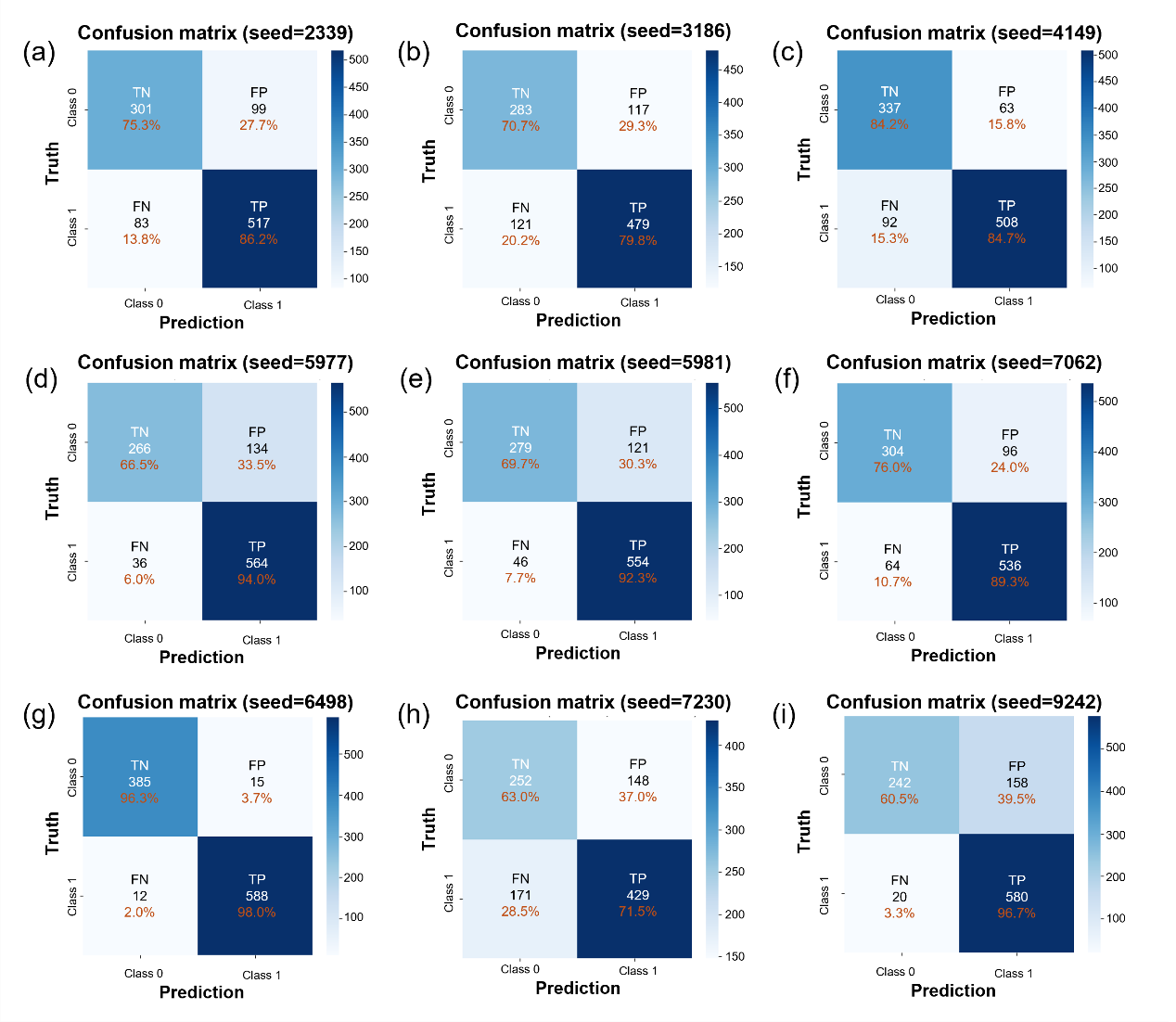


**Fig. S3** Confusion matrix of the best testing result under the seed-based data splitting scheme on an unavailable dataset.

**S4. Performance Comparison Across Different Classifiers**

To investigate the impact of different classifiers on the accuracy of the deep learning model, we adopted ResNet18 for image processing and feature extraction. We then replaced the fully connected (FC) classifier in our model with several commonly used alternatives, namely, Support Vector Machine (SVM), Random Forest, k-Nearest Neighbors (k-NN), Logistic Regression, and XGBoost for comparative evaluation.

The performance of each classifier was evaluated on the unavailable dataset using the same ten random seed partitioning schemes as described in the main text. We compared their average accuracy, true positive rate (TPR), false positive rate (FPR), true negative rate (TNR), and false negative rate (FNR), as summarized in Table S1. The results indicate that, under the ResNet18 feature extraction framework, these classifiers exhibit comparable classification performance, with average accuracy around 82%. Based on this comparison, we selected the relatively better-performing fully connected layer as the classifier in our final model.

## Table S1 Performance comparison of different classifiers based on ResNet18

| **Classifier** | **Accuracy** | **TPR** | **FPR** | **TNR** | **FNR** |
| --- | --- | --- | --- | --- | --- |
| **FC** | 82.5% | 88.1% | 25.9% | 74.1% | 11.9% |
| **k-NN** | 82.4% | 85.5% | 22.2% | 77.8% | 14.5% |
| **SVM** | 82.4% | 86.7% | 23.9% | 76.1% | 13.3% |
| **Random Forest** | 82.3% | 85.3% | 22.3% | 77.7% | 14.7% |
| **Logistic Regression** | 82.2% | 84.2% | 20.9% | 79.1% | 15.8% |
| **XGBoost** | 82.3% | 86.2% | 23.5% | 76.5% | 13.6% |

**S5. Performance Comparison of Feature Extraction Using ResNet18 and PCA**

Based on the findings from the *Performance Comparison Across Different Classifiers* section, we observed that the choice of classifier had a relatively minor impact on the overall model accuracy. To further investigate the effect of the feature extraction method, we replaced the ResNet18-based feature extraction framework with Principal Component Analysis (PCA) and evaluated the classification performance accordingly. Specifically, we extracted features using PCA and applied six commonly used classifiers: FC, SVM, Random Forest, k-NN, Logistic Regression, and XGBoost. We assessed their performance on the unavailable dataset using ten different random seed partitioning schemes. The average accuracy, TPR, FPR, TNR, and FNR across these tests are summarized in Table S2.

## Table S2 Performance comparison of different classifiers based on PCA

| **Classifier** | **Accuracy** | **TPR** | **FPR** | **TNR** | **FNR** |
| --- | --- | --- | --- | --- | --- |
| **FC** | 56.9% | 70.0% | 62.7% | 37.3% | 30.0% |
| **k-NN** | 60.0% | 99.4% | 99.1% | 0.9% | 0.6% |
| **SVM** | 57.1% | 70.3% | 62.7% | 37.3% | 29.7% |
| **Random Forest** | 61.1% | 92.1% | 85.5% | 14.5% | 7.9% |
| **Logistic Regression** | 56.8% | 70.0% | 62.9% | 37.1% | 30.0% |
| **XGBoost** | 55.3% | 69.6% | 66.2% | 33.8% | 30.4% |

ResNet18 serves as a learnable deep feature extractor, while PCA is applied directly to the input image data to produce reduced-dimensional feature representations. After PCA-based feature extraction, classification was performed using six different classifiers to evaluate the effectiveness of the resulting features. According to Table S2, the classification accuracy based on PCA features consistently hovered around 58% with a standard deviation of approximately ±3% across different classifiers. In contrast, as illustrated in Table S1, features extracted by ResNet18 led to significantly higher classification performance. These results highlight the superiority of hierarchical feature learning in deep neural networks over purely linear dimensionality reduction methods such as PCA.
